# Working across species down on the farm: Howard S. Liddell and the development of comparative psychopathology, c. 1923–1962

**DOI:** 10.1007/s40656-018-0189-y

**Published:** 2018-02-07

**Authors:** Robert G. W. Kirk, Edmund Ramsden

**Affiliations:** 1 Centre for the History of Science, Technology and Medicine (CHSTM), Faculty of Biology, Medicine and Health, University of Manchester, CHSTM Simon Building, Oxford Road, Manchester, M13 9PL UK; 2 School of History, Queen Mary University of London, Arts 2 Building, Mile End Road, London, E1 4NS UK

**Keywords:** Psychopathology, Animal model, Pavlov, Psychiatry, Experiment

## Abstract

Seeking a scientific basis for understanding and treating mental illness, and inspired by the work of Ivan Pavlov, American physiologists, psychiatrists and psychologists in the 1920s turned to nonhuman animals. This paper examines how new constructs such as “experimental neurosis” emerged as tools to enable psychiatric comparison across species. From 1923 to 1962, the Cornell “Behavior Farm” was a leading interdisciplinary research center pioneering novel techniques to experimentally study nonhuman psychopathology. Led by the psychobiologist Howard Liddell, work at the Behavior Farm formed part of an ambitious program to develop new preventative and therapeutic techniques and bring psychiatry into closer relations with physiology and medicine. At the heart of Liddell’s activities were a range of nonhuman animals, including pigs, sheep, goats and dogs, each serving as a proxy for human patients. We examine how Pavlov’s conceptualization of ‘experimental neurosis’ was used by Liddell to facilitate comparison across species and communication between researchers and clinicians. Our close reading of his experimental system demonstrates how unexpected animal behaviors and emotions were transformed into experimental virtues. However, to successfully translate such behaviors from the animal laboratory into the field of human psychopathology, Liddell increasingly reached beyond, and, in effect, redefined, the Pavlovian method to make it compatible and compliant with an ethological approach to the animal laboratory. We show how the resultant Behavior Farm served as a productive “hybrid” place, containing elements of experiment and observation, laboratory and field. It was through the building of close and more naturalistic relationships with animals over extended periods of time, both normal and pathological, and within and outside of the experimental space, that Liddell could understand, manage, and make useful the myriad behavioral complexities that emerged from the life histories of experimental animals, the researchers who worked with them, and their shared relationships to the wider physical and social environments.

## Introduction

On the death of Ivan Pavlov in 1936 the American psychobiologist Howard S. Liddell (1895–1962) was asked to explain to the readers of the *Cornell Daily Sun* the importance of Pavlovian science. Liddell described how he was building on Pavlov’s work “to bridge the gap between the causes of human and of animal insanity” (Anon 1936: 1). The following year, Cornell University invested heavily in Liddell’s Pavlovian research group. A 110-acre farm was acquired to allow “[d]eviations from normal behavior in the animals [to] be studied in their relationship to problems of delinquency and serious mental disorders” (Anon 1937: p. 6). Liddell’s experimental investigations of nervous disorders in animals, generously funded by the Rockefeller and Josiah Macy Jr. Foundations, were “expected to play a large part in the understanding and prevention of mental diseases in human beings” (Anon 1937: p. 1). Subsequently known as the Cornell Behavior Farm, Liddell established the leading American research group studying mental illness from a comparative and experimental perspective, respected worldwide for the “range, novelty and volume” of its work (Broadhurst 1960: 731). Extending Pavlovian techniques to create and investigate pathological mental states in diverse animal species, including dogs, rats, pigs, sheep, and goats, the aim was to establish stable experimental forms of neurosis for each species which, in turn, would serve as tools to develop new psychiatric therapies for human psychopathology.

While a commitment to comparative psychopathology was at the heart of Liddell’s work, this is not to say that bridging the gap between human and animal neurosis was widely accepted. On the contrary, the question of whether nonhuman species could be said to exhibit mental illness, or what came to be known as “experimental neurosis”, was as hotly contested by psychoanalysts as it was assumed by Pavlovian and psychobiological experimentalists. Following Freud, psychoanalytically inclined American psychiatry strenuously objected to comparative studies of mental illness across species. Neuroses were human, consisting of:

psychic attitudes which we can understand only if we study human beings from the psychic point of view. There is … no hope of our finding bodily interpretation of the neurosis from this point of view. Many important relationships between body and mind may be studied and clarified by the method of Pavlov … but not the central problem of the neurosis (Schilder 1929: 516).

Pavlov robustly contested such views, arguing that the comparative studies of neurosis were not only legitimate but necessary. Scientific understanding could only be obtained by “an analysis of a more complex phenomenon in terms of more elemental and simple phenomena”. Accordingly, “neurosis in man must be … understood … with the help of studies of neurosis in animals, which are naturally more simple” (Pavlov 1932: 1012).^1^ Liddell, who had visited Pavlov’s laboratory, saw the Pavlovian approach as a means to make a science of psychiatry. Moreover, he believed the “physiological tradition and this psychodynamic tradition will eventually coalesce … when it is recognized that between the organic position and the psychodynamic position, there is another distinct viewpoint—the behavioral or psychobiological position”.^2^ Liddell’s distinctive experimental program, which placed comparative methodology at its center, was designed to bring about the rapprochement of Freudian and Pavlovian schools of thought.^3^

This paper examines Liddell’s attempts to work across disciplines and species in the pursuit of a scientific understanding of neurosis. However, as the work progressed, and the more experimental control Liddell exerted over his animals, the more he became aware of importance of the complex relationship among and between organisms and environments. Liddell, like other students of experimental neurosis, was far removed from the “staunch behaviorist” that he and others working with the Pavlovian method are often described (Rodkey 2015: 118). He recognized the necessity of understanding the individual animal’s biological and social needs. Over time, he increasingly drew the wider environment into his experimental system to help distinguish normal from pathological behavior. Most of all, he learned to interpret animal behavior in a way that lent itself to traveling beyond the confines of the experimental laboratory and into the worlds of clinical medicine, psychiatric practice and human society.

By exploring the ways in which Liddell sought to unite the places and practices of experimental laboratory and clinical field, we draw from articulations of ontological hybridity as an analytic tool. In addition to disrupting simple dichotomies, a characteristic of this literature is an attempt to challenge the privileged space of the human subject as agent of historical change by blurring the boundaries between human and animal, nature and culture, biology and environment.^4^ Accordingly, we argue that Liddell’s Behavior Farm can best be understood as a “hybrid” place. Liddell’s farm resembles the amalgam of vivarium, biological farm and field station that Robert Kohler identified in his study of laboratory and field relations; a place where biologists sought to bring the natural world inside to study under controlled conditions. The Behavior Farm provided Liddell with a domesticated space which resembles what Kohler described as a core requirement of experimental knowledge production: an “artificial and ecologically simplified environment”. For Kohler, experimental production demanded an invariable, placeless and predictable site stripped of the complexity and uncertainty of the field (where observation must be “flexible and opportunistic”) (Kohler 2002: 18). However, while not a wild or natural place, the Behavior Farm was nevertheless far from placeless or predictable. Animals roamed freely in their ‘natural’ environments of pastures and woodland; whilst the designated laboratory space was, likewise, far from invariable. For Liddell, behavior in one environment could only be understood through reference to the other, leading him to make an experimental virtue of what Kohler designates as the “second-best practices” of “observation and comparison” (Kohler 2002: 3). Contrary to Kohler’s implicit assertion that movement of concepts and methods follows a path from laboratory to field, we show how Liddell’s experimental system on the Behavior Farm worked across both sites (Kohler 2002: 61).^5^ We show how Liddell increasingly turned to the concepts and methods of ethology: a discipline that Kohler characterizes as “an outdoors science” (Kohler 2002: 134).^6^ For Liddell, ethology offered a means to understand holistically the complex relations between individual animals and their laboratory surroundings on the one hand, and, on the other, their social lives outside in the fields and pens of the farm. We conclude by suggesting that the Cornell Behavior Farm was an extremely creative “border zone”, a place where concepts and practices of laboratory, clinic and field coalesced to produce a productive and influential comparative psychobiology.

## Liddell’s early comparative research at Cornell: working across body and mind

In 1918, having graduated from the University of Michigan with a master’s degree in psychology, Liddell entered Cornell as a doctoral student in physiology working under the Scottish trained physiologist Sutherland Simpson. Simpson, who had worked closely with Edward Sharpey-Schafer at Edinburgh, was engaged in an extensive study of endocrinology focused on the thyroid and parathyroid pathologies.^7^ Through the 1920s, at Simpson’s Physiological Field Station, Liddell was tasked with studying fundamental physiology in the form of thyroid function (Liddell 1958: 245). Thyroid glands were removed from young sheep and goats to assess the effect on growth and physiological functions such as body temperature and heart rate. Simpson had chosen to work with sheep and goats for multiple reasons: they were inexpensive to maintain, grew to a convenient size and much was known as to their genetics, nutritional needs, and susceptibility to diseases (Liddell 1954: 49). Surgical removal of the thyroid was easily accomplished in ruminants without disturbing the external parathyroid. Most important, however, was that these species frequently gave birth to twins of the same sex, an ideal pairing of an experimental and control animal.^8^ Once thyroidectomized, animals were found to be dwarfed in size and often exhibited muscular weakness, lethargy, and an apparent “dull, stupid appearance” (Liddell 1927: 41). Liddell’s early work explored the physical effect of thyroidectomy on spontaneous activity. Having established that thyroidectomized animals often became so weak as to be unable to walk, he sought substances, such as thyroid extract, that would alleviate the condition (Liddell and Simpson 1925). Thyroidectomy served as a crucial tool within an experimental system for the investigation of thyroid deficiency, intended to provide a greater understanding of the condition and to develop new therapies.

**Fig. 1:**
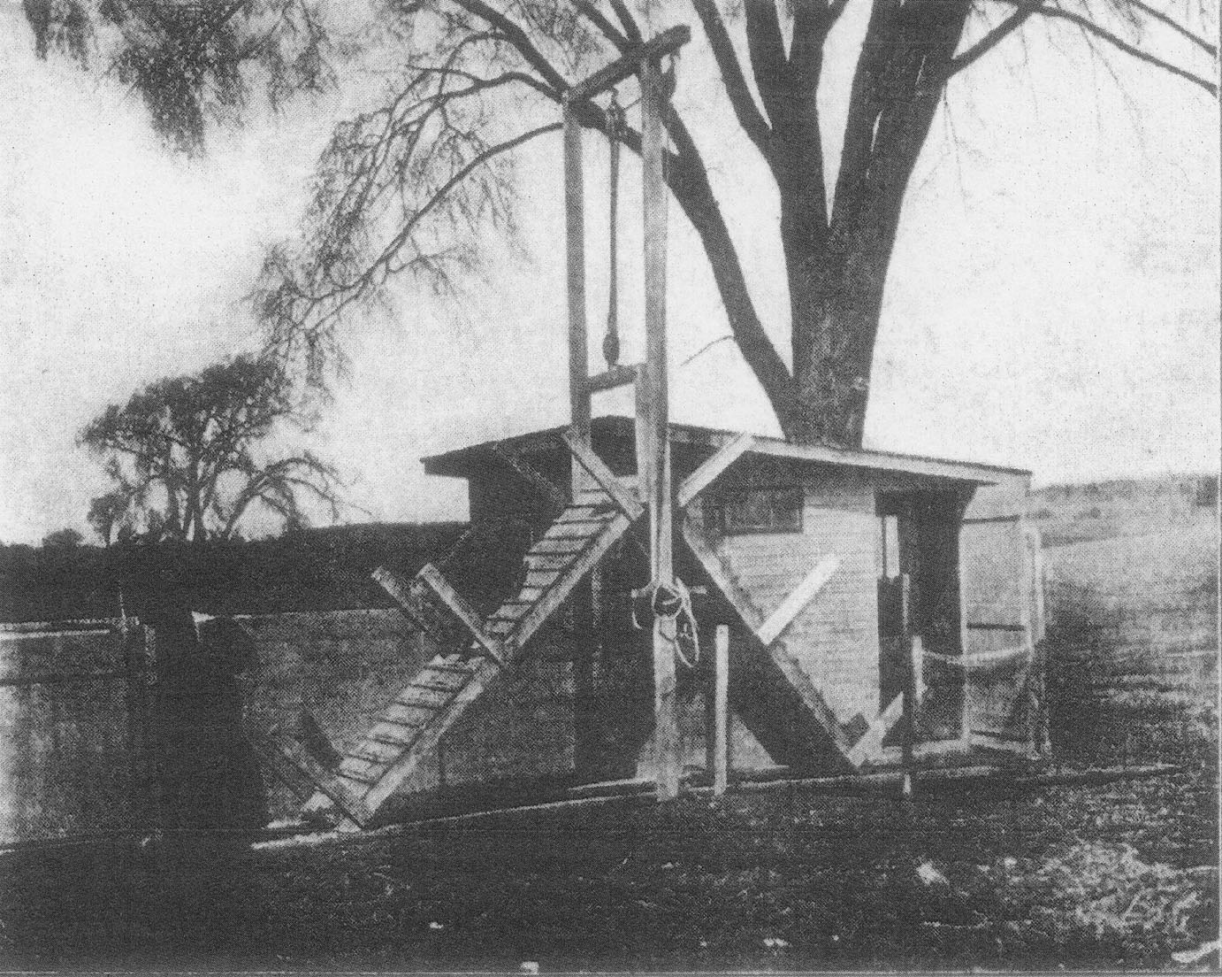
Liddell’s experimental apparatus for quantifying muscular weakness, from Liddell (1923: 192)

The use of “spontaneous movement” as an indicator of physiological normality was made possible by Liddell’s use of a pedometer, fastened to the foreleg of an animal via a snug leather pocket belt. This approach bore the hallmarks of Liddell’s experimental approach, not least his valorization of the “quantitative method” and his use of technological innovation to allow the animals to act more freely. However, it also embodied a generative tension at the heart of his work: between the scientific ideal of objective quantitative data and the inescapable necessity of subjective observational knowledge of animals both as individuals and as species. In the case of the thyroidectomized animals, Liddell encountered a problem: their smaller frames meant they often took more steps to cover the same ground. While this could be partly controlled through measurement and the individualized calibration of the pedometer, adapting the procedure to measure muscular weakness rather than merely quantifying degrees of lethargy, proved particularly challenging. Consequently, one had to understand sheep and goat behavior to properly interpret the quantitative data. A thyroidectomized sheep, even with severe muscular weakness, could match its normal twin in the context of a slow-moving grazing flock. Diminished muscular power revealed itself only when the flock was forced to move at speed, such as when driven to the pen. Accordingly, Liddell designed an experimental apparatus consisting of an adjustable inclined plane. Sheep could be induced to ascend the apparatus by using the “flock instinct” whereby the group would follow the lead. Adjusting the incline provided a means to measure muscular weakness as thyroidectomized sheep would eventually be unable to follow the herd, despite trying until exhausted (Fig. 1).

In this way, the tension between the scientific ideal of objective quantified data and the necessity of subjective observational knowledge was generative of new ways of working with and producing comparative knowledge.

**Fig. 2:**
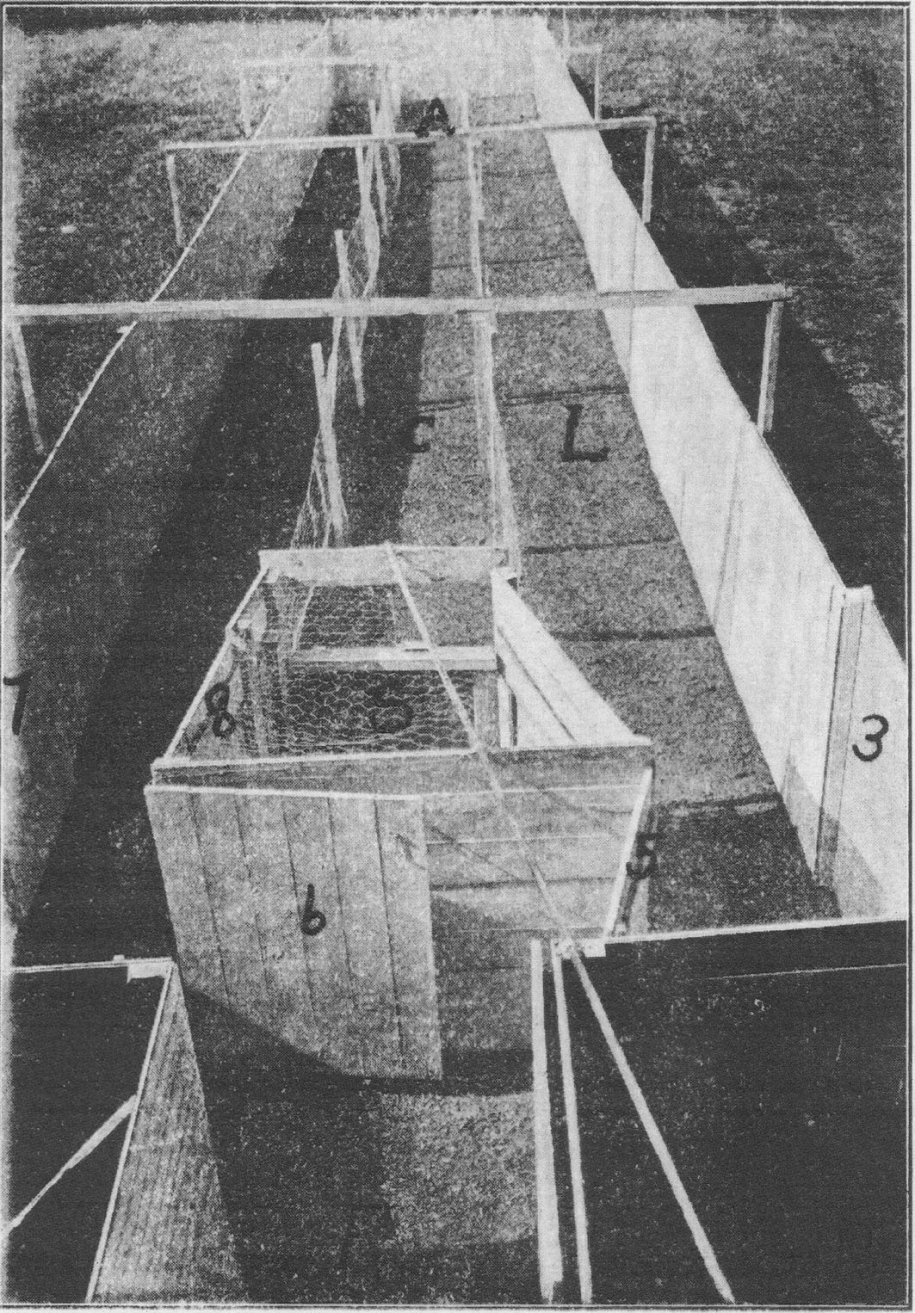
Liddell’s sheep labyrinth, from Liddell (1923: 195–196)

**Fig. 3:**
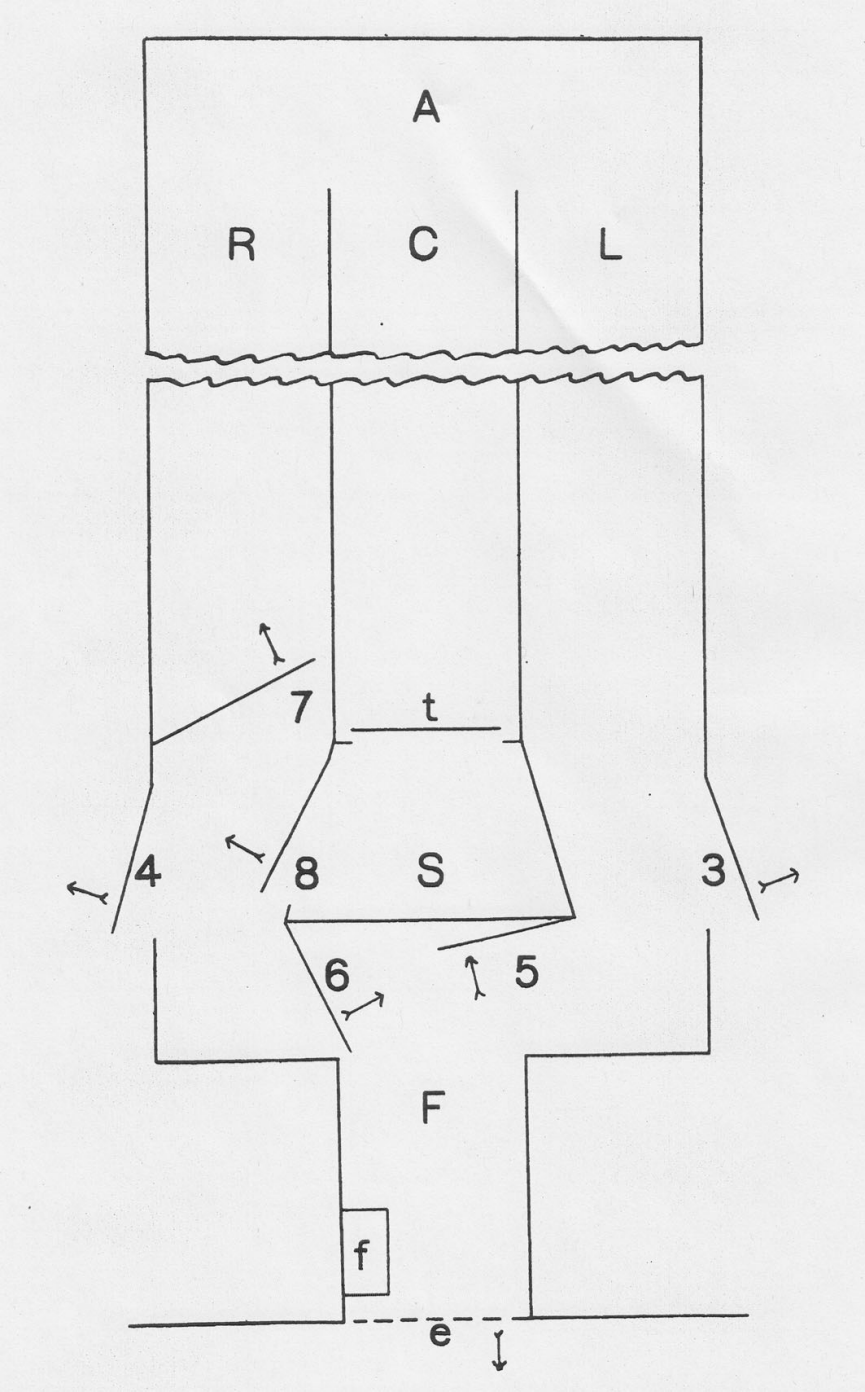
Liddell’s sheep labyrinth, from Liddell (1923: 195–196)

The conditions of scientific knowledge were a central concern for Liddell who maintained a strong sense of epistemological humility. When Liddell abandoned his early commitment to psychology in favor of studying physiology at Cornell, he did so because he saw the latter as having more rigor and credibility as a science. Simpson, however, was to make a virtue of Liddell’s prior experience, pushing him to address the issue of whether the sheep’s mentality had also been blunted in a comparable way to that of humans who suffered thyroid deficiency (Liddell in Tanner and Inhelder 1956: 18). Liddell was initially wary of comparative questions that addressed psychology, knowing full well that “[c]onclusive statements as to the effect of extirpation of the thyroid glands on the intelligence of an animal cannot be based merely on chance observations” (1923: 194). Accordingly, Liddell again developed a quantitative approach, this time adapting techniques for investigating animal intelligence and learning in the rat (Figs. 2, 3).

Liddell constructed large out-door labyrinths with three parallel alleyways, adjustable gates and moveable dead ends. They measured 67ft in length, 10ft in width, and 6ft in height, requiring the researcher to observe from the height of a second story building (Fig. 1).

A sheep was placed in the start position (s) and was expected to locate a compartment (f) where the animal could eat food and view the flock. Liddell was aware of the difficulty in establishing and maintaining an animal’s co-operation, without which the validity of any measurement would be undermined (as the drive to complete the labyrinth had to be constant). While there was good reason to suspect the drive for food could diminish with time, fluctuate with appetite, or be altered by the thyroidectomy, Liddell believed the “flock instinct” would ensure a consistent drive to navigate the labyrinth. The progress of individual animals was timed and a pedometer used. Behavior and errors in retracing a known path were recorded by Liddell, observing from a concealed window on the second story of a local building (from where he could release the animal using a pulley system). Once the sheep had learned the labyrinth, successfully navigating the path to F a set number of times without error, reactions to environmental change could be measured through altering the maze.

Three years of laborious work observing sheep in mazes revealed “[s]ignificant differences in maze behaviour between the thyroidectomised animals and their twin controls … but it was found impossible to interpret these differences by using the concepts of animal psychology” (Liddell 1927: 50). Practical problems included the objective definition of “error”—initially including many pauses and instances of “loitering”. However, once an animal was familiar with the labyrinth, loitering became the norm. This increased the error count and the time taken to complete a run. Therefore, the category of “error” accumulated a degree of interpretive flexibility. Sheep were also prone to pausing to investigate and consume weeds, grass, spilt feed or other novel objects, necessitating the extensive cleaning and weeding of the labyrinth each morning. However, no matter how the experimental set up was adjusted, the behavior of the sheep could not be made to correlate with the hypothesized dullness of mentality expected from thyroidectomy (or any other theory for that matter):

One of the cretins [thyroidectomized sheep], tested during a period of extreme lethargy 147 days after the thyroidectomy, failed to escape from the maze. On the first trial it entered the cul-de-sac, from which it was removed after two hours. On the two following days it did not leave the central alley, and was removed after periods of two and two and a half hours respectively. This result seemed to indicate decreased intelligence following thyroidectomy, since the normal twin readily relearned the maze in six trials with a total of eight errors. In striking contrast, however, were the data from the other lambs … In every case the cretin relearned the maze with fewer errors than the control… We have demonstrated retention of this simple maze habit in thyroidectomised sheep and their controls after an interval of two years without discovering any significant difference between them as regards the time necessary to perfect the old habit (Liddell 1927: 42).

There appeared to be no discernible difference in intelligence and learning ability between normal and thyroidectomized animals. Liddell’s research had reached an apparent dead end.^9^ Yet, considering the effect of the thyroid on human intelligence, it seemed implausible that such demonstrable physiological difference had no effect on mentality. Many years later, Liddell lamented that the problem was not with the comparative hypothesis but rather with the “sheep’s insistence on running through the maze for its own reasons—not for the reasons or ‘motives’ that we attributed to it” (Liddell 1954: 51). While food and the flock instinct were significant drives, months of fruitless observation of inconsistent behavior led to the suspicion that sheep possessed their own hidden and individualistic “ambitions” for running the labyrinth.^10^

Liddell explained the failure in terms of not having sufficient control of the experimental animal or environment. With the labyrinth, Liddell was rendered a passive observer; it was the sheep who determined whether they would conform to the demands of the experiment or pursue other interests and activities. He wrote of his growing irritation and frustration. On one cold winter’s day, a lethargic sheep chose to lie down for an hour before eventually choosing to complete the labyrinth run, while Liddell stood observing and shivering behind his cold barn window. Accordingly, Liddell sought a more precise measure of learning. This was provided when G. V. Anrep (a former assistant to Pavlov) visited Cornell in 1923 to lecture on Pavlovian techniques. Liddell recognized the potential of the conditional reflex for overcoming the problem of studying mental processes:

It was not without intention that I used the adjective “so called” in speaking of “mental processes”. If a *complete analysis* of the psychical faculties of the higher animals be set as a problem for an investigator, he has no right to speak of such faculties as existing in these animals, and in fact he cannot do so without being unfaithful to the principles of natural science… If he (the investigator) were to speak of the psychical faculties of the higher animals he would be transferring ideas from his own inner consciousness to natural phenomena with the result that there would be a repetition of what men did when they first turned their thoughts to the consideration of nature and attributed to inanimate objects their own intelligence, will, and sensations. For a consistent investigator there is in the higher animals only one thing to be considered—namely, this, that or the other external response of the animal to external impressions (Pavlov 1906: 911).

In now seemed apparent that the labyrinth was far too complicated a system to ever provide a definitive statement of the effect of thyroidectomy on sheep intelligence. Liddell had to narrow down the scope of his work to something approximating the simplicity of Pavlov’s salivary reflex. Under the direction of Anrep, in 1923 Liddell established the first American laboratory arranged around the Pavlovian method and set about applying the conditioned reflex to the study of mental dullness in normal and thyroidectomized sheep.

As cud-chewing animals, sheep salivated continuously, ruling out a direct transposition of Pavlov’s study of salivary reflexes with dogs. Instead, Liddell worked on a conditioned motor reflex. By restraining a sheep in a harness on a platform and then mildly shocking the foreleg with electricity, timed and signaled by the clicking sound of a metronome, Liddell found the animal could be conditioned to respond by lifting its foreleg from the floor. He could also measure respiration on smoked paper kymograph: “Such graphic registration made me feel that I was once more back in physiology having escaped from the maze of psychological speculation” (Liddell 1958: 248). Finally, Liddell had found a means by which the researcher, as opposed to the animal, was in control of the experiment. However, this new precision revealed that there was no measurable difference between normal and thyroidectomized animals; both displayed the same capacity to acquire and maintain the conditioned reflex, and neither proved more adept at discriminating between different metronome rates. This second failure to demonstrate the validity of comparative reasoning could have brought his work to an end, had it not been for a “laboratory accident” which fundamentally altered the trajectory of his research.

In 1927, Liddell had presented a pair of twinned sheep with a challenging task—establishing delayed conditioned responses whereby the signal of shock was timed for the sixth click after an initial click. After several days, both sheep were struggling to display a stable response—raising a foreleg on the 4th, 5th, and only occasionally on the 6th. Impatient to meet a conference deadline, Liddell intensified the conditioning schedule, doubling the daily work of each sheep. The following day, the normal twin became highly excited when brought to the laboratory, struggling violently at the sound of the metronome and exhibiting small, jerky movements of the trained foreleg which Liddell likened to the twitching of human facial muscles in those afflicted by a tic. In contrast, the thyroidectomized twin continued as before, willingly entering the laboratory and eventually displaying the desired delayed reflex even when the schedule was doubled for a second time. Whereas the normal twin had become so alarmed as to refuse to enter to the laboratory, the thyroidectomized animal continued its work quietly and without complaint. Here at last was a clear difference: thyroidectomized animals suffered not dullness of intelligence, but dullness of emotion (Liddell and Bayne 1927). The normal twin was exhibiting all the signs of emotional breakdown that Pavlov had described as an “experimental neurosis”. Bolstered by the quantitative experimental method made possible by the Pavlovian conditioned reflex, Liddell overcame what he later described as his “squeamishness about anthropomorphizing” and embarked on the comparative study of emotion (Liddell in Tanner and Inhelder 1956: 18). The path to a comparative psychopathology opened before him.

## Making a science of psychiatry: the farm as a hybrid space

“Neurosis”, Freud (1939: 94) had written, “seems to be a human privilege”. Liddell set out to demonstrate, experimentally, that this was not the case. His attempts to exert increased experimental control over his animals had resulted in the accidental discovery of severe agitation in the sheep that appeared to mirror Pavlov’s description of ‘neurosis’ in the dog. While for some of his contemporaries, such as the neurologist and psychiatrist Kurt Goldstein, such errant behavior showed the conditional reflex to be a destructive and even “catastrophic” imposition of a “routine drill” (1939: 177–178), it could also serve, for this very reason, as a useful basis for a new comparative psychopathology. Liddell became a leader in the rapidly growing field of ‘experimental neurosis’, even being described as the “Father of American Experimental Psychopathology” (Block 1963: 2). He was joined by an array of physiologists, psychiatrists and psychologists who were using Pavlovian techniques on a variety of animals, from rats to monkeys, as a means of rigorously testing, advancing or displacing, psychoanalytic approaches to mental disturbance and developing a new and more objective scientific basis for studying and treating mental disorder. In the place of questionable memories and verbal responses, were the conditioned reflexes: a means of interrogating psychical life objectively in the laboratory through precise quantitative measures of muscular movements and secretions. The reflex was the mechanism by which an organism continually adjusted to a changing environment, substituting new reactions for old, and constantly building upon the foundation of the unconditional reflexes which fulfilled the basic requirements of an organism. What Pavlov described as “higher nervous activity” was to be understood physiologically, as the combined processes of inhibition and excitation seated in the cerebral cortex.

Just as the reflex method had provided Liddell with more control over his experimental subjects in his study of thyroidectomy, by submitting the animal to stress in “graded” doses of “graded severity” in the study of psychopathology experimenters could now determine and measure the point at which the animal’s adaptive responses became overwhelmed and broke-down.^11^ They could then examine the consequences of such a breakdown over time, often for years after the event, and attempt, using various methods of training and drugs, to ameliorate the symptoms. As Horsley Gantt at Johns Hopkins declared, the ability to create, trace and influence the development of a breakdown was far more useful “than to behold the full-fledged neurosis”, and was “something that we are unable to do in the clinic” (Gantt 1944: 38). Drawing from Pavlov and Gantt, Liddell adapted their technique of “difficult differentiation” to produce a conflict between excitation and inhibition in the nervous system: applying two signals that became increasingly similar, one followed by food (excitation), the other with none or by a mild electric shock (inhibition), or simply switching the signal and stimulus (the signal for food, now resulting in a shock). Liddell found the consequences of such seemingly innocuous changes to be profound, producing an “amazingly vehement response”—the animal struggled, its breathing became irregular, its heart rate increased, it lost all powers of differentiating between signals, and it would often staunchly refuse to reenter the experimental chamber.^12^

**Fig. 4:**
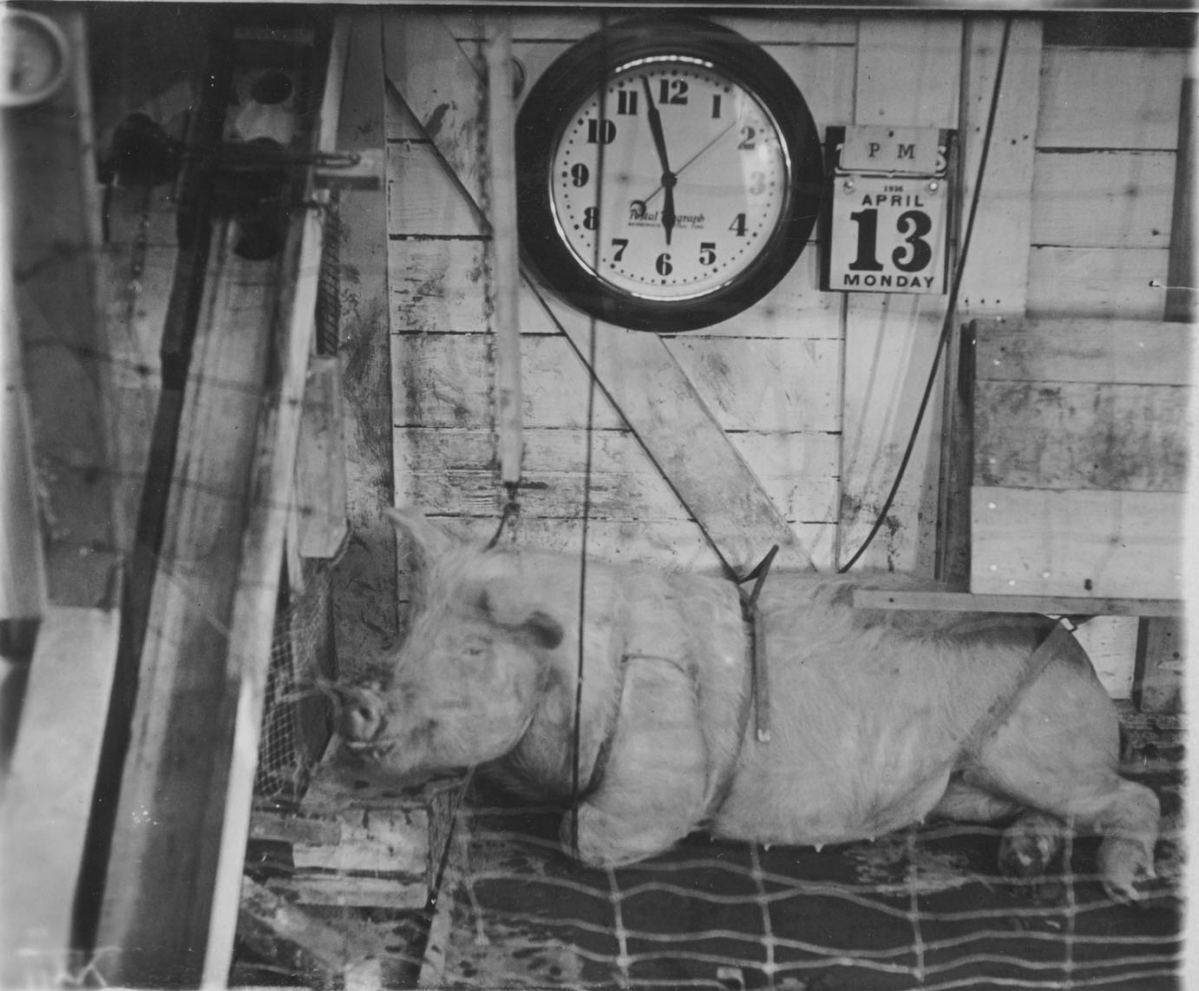
Experimental neurosis in the pig. The animal learned to distinguish between two tones, one for shock and one for food, which signaled to the animal that it could open the food-box (bottom left) and receive a piece of apple. They alternated the signal and stimulus, the tone for food now resulting in shock, and exerted unwelcome restraints such as shocking the pig when it touched the fence or the food box without the signal being given. This resulted in neurotic behavior whereby the animal would refuse the apple in the experimental room, even when freely given, and exhibited tantrums and extreme aggression—attacking the food-box, the pen, and even the experimenters. Image courtesy of Division of Rare and Manuscript Collections, Cornell University Library, HSL, Box 10

Where Liddell differed at this stage of his research with Pavlov and Gantt’s approach was with regards to the choice of animal. While he agreed that the “mentally gifted” monkey was too independent, wild, “erratic and fidgety”,^13^ the dog was so eager to please and emotionally attached to the observer it would lead to experimental bias.^14^ Farmyard animals, he argued, were ideal. As domesticated animals, they were comfortable in the presence of the human, yet they were also indifferent to the experimenter as they had no emotional attachment, reducing the possibility of observer-expectancy effects. The varieties of species and breeds could also capture different aspects of human personality types: “sheep appear to be timid, apprehensive and gregarious, while the pig is characteristically individualistic, and aggressive and exhibits tantrum behavior” (Fig. 4).^15^

Liddell’s success in generating “neurosis” was rewarded by Cornell University, the Rockefeller Foundation and the Josiah Macy Jr. Foundation. In 1938, he moved from the Physiological Field Station, which he described as a “modest farm laboratory” of 9 acres with a “small barn and a single-storey wooden laboratory and storage building with adjoining runways for the animals”, to a 110-acre farm with a newly constructed laboratory for teaching and research (Liddell 1958: 245).^16^ This Liddell christened the Behavior Farm Laboratory. He now had much-needed space and equipment to expand his activities. He could maintain diverse breeds and species to establish the importance of heredity, constitution and temperament in nervous disorder. While he conceded that the pig “proved to be our master”, becoming too dangerous to work with, it was replaced with the goat, a similarly independently-minded animal and a useful contrast to the more docile sheep, which “likes to be a slave”.^17^ Others at the farm also worked on generating neuroses in dogs and rodents, a means of establishing the common features of neurotic behavior across animal species. Liddell built a multi-disciplinary and collaborative research and teaching enterprise, bringing together psychology, physiology, biochemistry, zoology and various other biomedical and behavioral fields, and research scientists and clinicians. To bring some coherence to such diversity while capturing its breadth, Liddell increasingly adopted the term ‘psychobiology’—a psychology that was grounded in the experimental biological sciences.

By 1952, he could boast of an experimental flock of 45 sheep and 100 goats. This large number of animals allowed him to explore emotional reactions in relation to age and sex through the “long-continued repetition of a few standardized emergency situations”.^18^ He could also alternate—testing similar animals, including twins, with different variations of the conditional reflex technique. This allowed them to identify which aspects of the procedure were particularly damaging to the animal and generate different forms of neurosis. For example, a conditioning regime involving a brief shock to animal’s foreleg, resulted in a “new type” of withdrawn animal with “neurotic rigidity” of trained limb resembling “conversion hysteria in the human subject”. This condition differed in its characteristics from those of the intensely active and agitated neurotic animal.^19^

Most importantly, with such large amounts of space and numbers they could now compare the experimental animals with other normal controls in the flock. This allowed them to address growing questions regarding the distinction between normal and pathological behavior. Liddell was acutely aware of the fact that being forcibly isolated in the sterile environment of the laboratory and continuously prodded and poked by researchers, was unnerving to the animal. He also recognized the laboratory to be far-removed from the “stripped down-simplicity” characterized by Kohler (2002: 7), describing the animal as “almost buried from sight in a thicket of straps, electrodes, cables, levers, recording pens and kymograph paper”.^20^ Consequently, he began to make the setting more natural and less alarming, allowing a more precise focus on the timing of stimulus and its emotional effects: “The conventional Pavlov frame with its restraining straps was first to go”.^21^ Having stripped away much of the experimental apparatus, the animal would now enter a bare room from the barnyard, to be observed through a one-way screen and the stimulus administered by a very light cable suspended from the centre of the ceiling: “The laboratory room soon becomes, then, just a corner of the barnyard and pasture”.^22^ These alterations had immediate rewards in providing more consistent data across different animals, Liddell describing how much of the animal’s distress in their earlier work seemed to have resulted from their own tension and irritability as experimenters carrying out by hand the monotonous and stressful delivery of the training stimuli while taking notes and keeping the recording equipment going: “in our former studies the seeming range of individual susceptibility of sheep and goats to experimental neurosis was probably a more reliable measure of the range of individual temperaments among the investigators”.^23^

At the same time, the farm also allowed Liddell to follow the animal into their ‘normal’ environment to observe and demonstrate the permanent effects of the emotional trauma experienced in the laboratory. They could witness the behavior of neurotic animals in the pasture, finding them to have lost their normal gregariousness, becoming easily alarmed and running in different directions to the rest of the flock: “Each of our neurotic animals has his own individual system of worries”.^24^ In one example, a goat named “Sonya” was described as “fearful”, “an introvert”, refusing to graze with others, ill at ease in the herd, and treated as a “stranger”: “Her survival value is much retarded; her behavior does not resemble the gregarious pattern of her own species”. When other animals went into the barn, she stayed alone: “The most important aspect of her is the fact that she does not show any orientation to other animals”.^25^ They also followed the animals into the barn:

Several years ago we published definitive evidences that the neurotic sheep takes its worries home to the barn at night… The animal exhibits diffuse agitation in the laboratory, with frequent and vivid startle reactions, laboured breathing, and rapid irregular pulse. Even weeks or months after the tests have been discontinued the animal exhibits its perturbation in the barn at night. With the aid of a long-distance stethoscope the observer, in a shed outside the barn, can listen to the heart sounds of both normal and neurotic sheep … [T]he normal sheep’s heart beats slowly and regularly; by contrast, the neurotic sheep’s heart may be beating twice as fast with wide fluctuations of rate and with frequent premature beats (Liddell in Tanner and Inhelder 1956: 127).

**Fig. 5:**
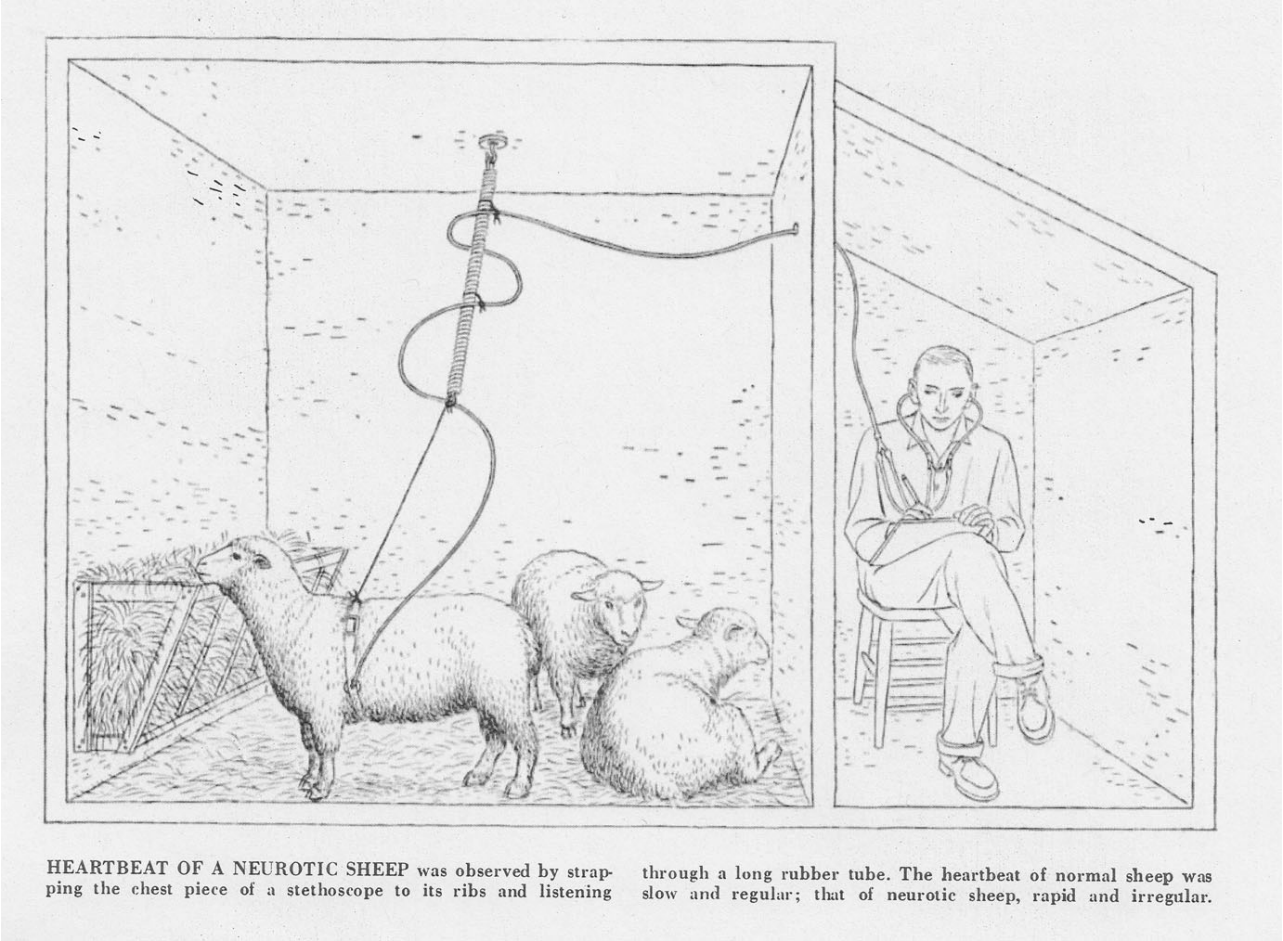
Observing a neurotic sheep (Liddell 1954: 56)

By blurring the boundary between laboratory and field—that is, by taking some of the experimental equipment into the barn, observing the animals in the pasture, and making the experimental space more like the barnyard—Liddell was using the hybrid status of the farm to his advantage. He could respond forcefully to growing criticism of the experimental neurosis paradigm that suggested that so-called “neuroses” were merely situational fear or anxiety responses: which is to say originating in and confined to the laboratory, and thus of no real relevance to human psychopathology (Hebb 1947: 7). As he explained to one of the critics, “since the neurotic patterns which we have observed can be established on a truly chronic basis, a serious attempt must be made to explain them”.^26^ Understanding experimental neurosis was therefore a legitimate and important scientific endeavor, although he admitted, “At the present, I am frankly unable to do this”.^27^ In extending the scientist’s gaze beyond the laboratory walls, Liddell was arguing that the behavioral pathology of the sheep or goat was to be evaluated in the context of the animal’s normal day-to-day life, and, in this case, it was life on their natural habitat of the farm (Fig. 5).

His focus on the animal in the barnyard reflected a growing divergence from Pavlov’s physiology, to which he described himself as having been naively committed: “each time the animal came to the laboratory it became a laboratory preparation and I bid it good-bye at the door; my responsibility for it was done. But it was not that simple”.^28^ He also began to question Pavlov’s interpretation of neurosis in terms of a clash between the processes of excitation and inhibition. Pavlov’s method of difficult differentiation was, in fact, over-complicated and unnecessary. The very process of conditioning was destructive to the animal, irrespective of the combination of signals and stimuli applied. Even having stripped back much of the physical restraint, in the conditioning routine the animal was still forced to endure an unwanted process for an hour at a time, day after day, even year after year. Over time, as the animal realized that flight or fight was impossible, the restriction became psychological: “The sheep has no surcease from alarm. It is encased in a psychical strait jacket of anxious apprehension”.^29^ The animal became physically tense, emotionally muscle bound, and permanently vigilant: “Since all types of Pavlovian conditioning develop in the animal increasingly rigid control of its emotional reactions to danger *all conditioning is difficult conditioning* and will, if long continued, lead to emotional disaster” (Liddell 1956: 81).

In sum, Liddell reached the conclusion that “the conditioned reflex described by Pavlov is not primarily an example of ordinary learning nor a manifestation of intelligence. It is, instead, primarily a manifestation of the emotional context of behavior” (Liddell 1954: 55). In recognizing the conditional reflex as primarily emotional and always traumatic, a comparative scientific study of the neurosis now seemed entirely possible, even though:

[t]he sheep is not a man; nevertheless, we can freely empathize; we have our own view as to how we would regard the situation if we were in the animals’ place; and we have taken this matter seriously. Every single procedure which we apply to our animals in the Pavlovian situation we try on ourselves, and this has led to important clues (Liddell in Tanner and Inhelder 1956: 18).

Clues alone, however, were not a science. While the conditioned reflex provided a quantitative methodology, successful reasoning from animal to human necessitated an intimate subjective knowledge of the relationship between experimenter and experimental animal, and a comprehensive understanding of the animal in its ‘natural’ environment. More was required to bolster the credibility of Liddell’s comparative approach to psychopathology. How could one bridge the experimental practices of laboratory science and the intimate observational skills more usually associated with the field or clinic? Liddell found a solution in the emerging science of ethology.

## Comparative psychopathology on the behavior farm: working across disciplines

Liddell’s use of the conditioned reflex resonated with Pavlov’s promise that agency and desire could be “incorporated into flesh and blood, transformed from a subjective sensation into a concrete factor of the physiological laboratory” (Pavlov 1902, quoted in Liddell 1942: 390–391). While the object of study, emotion, had been rendered a “concrete factor”, this approach nevertheless depended on the experimenter’s personal experience of the animal as an individual built over an extended period. Liddell described this distinctive relational bond as a form of shared “intimacy” gained from months, even years, of collaboration (Liddell 1942: 391). Making an explicit virtue of intimacy was the characteristic which distinguished Liddell’s psychobiological approach from wider physiological studies of emotion.^30^ Whereas both relied on the “cooperation of the animal”, physiology failed to make a resource of subjective knowledge as “intimacy between animal subject and investigator is taken for granted and does not enter into the appraisal of the results of experiment” (Liddell 1942: 391). For Liddell, in contrast, managing intimacy was a guarantor of experimental knowledge “similar in fundamental respects to the rapport between patient and psychotherapist” (Liddell 1942: 394). In part, Liddell’s position followed from his distinctive understanding of conditioning as a “traumatizing procedure” as opposed to an “impersonal observational procedure”. Moreover, the approach of working with animals over their natural life spans meant that the “[t]he intimacy which develops between animal and experimenter during prolonged conditioning must enter into the appraisal of the results of the experiment” (Liddell 1942: 394–395). Accordingly, Liddell began to cautiously valorize the role of intimacy in the performance of conditioned reflex research. As his research program developed, therefore, a new and productive tension emerged between the desire for objective quantified knowledge and the intimated knowledge (or ‘case history’) of the individual animal.^31^

By the 1950s, Liddell’s writing had become more confident. He argued that studies of non-human animals could establish psychiatry as a truly objective scientific field. On the one hand, the conditioned reflex provided quantitative (‘objective’) evidence which could be replicated in the laboratory. On the other hand, the ability to interpret this evidence required an understanding of the individual animal within the context of its social and physical milieu, thus accommodating an individual case-based approach akin to that used in human psychiatry. Liddell’s early pursuit of objective physiological knowledge resulted only in him having repeatedly “received the strong impression that our experimental animal was trying to say: ‘Remember, you are not studying physiology, you are studying me!’”.^32^ Valorizing the individual allowed Liddell to present his work as responding to a growing interest in biological approaches to psychiatry. Stanley Cobb noted this trend in his influential textbook *Foundations of neuropsychiatry*:

Many psychologists and psychiatrists who deal with human beings become engulfed in the complexity of their material and never become acquainted with the simple and important facts of ‘natural history.’ Training in the simple biology of barnyard and forest is a great educational advantage. The fact that many leading psychiatrists are urban products, knowing little of these biological fundamentals, has led to much misunderstanding of what an instinct really is and to much vague use of such terms as ‘instinctual.’ (Cobb 1958: 253).

Mention of the ‘barnyard’ was an indirect reference to Liddell. Cobb, known as an advocate of psychosomatic medicine, was a regular visitor to Liddell’s Behaviour Farm.^33^ Liddell appropriated this trend not only by the conventional mechanisms of publication, presentation and the media, but by literally inviting psychologists, psychiatrists, psychoanalysts, and allied professionals “back to the barnyard” to observe the work of the Behavior Farm first hand.^34^ His aim was that on leaving, even the most committed Freudian would have come to accept that:

[t]he objective manifestations of experimental neuroses are basically the same for dog, pig, sheep, goat and man. The behavior of the neurotically ill individual, animal or man, is rigid, ineffectual, and unrealistic. It limits him in meeting his total life situation in its historical continuity. Patterns of frustration behavior persist of their own momentum year after year in sheep and goat and in man, decade after decade.^35^

By the close of the 1950s, Liddell’s Behavior Farm operated as a unique gathering point, spanning physiological, psychobiological, ethological and psychiatric studies of behavior.

While Liddell’s alignment of his experimental program with psychiatry established new potential for collaboration across multiple disciplines, it also raised significant epistemological challenges. How could one maintain psychobiology as a science when it depended on knowledge and experience of intimate personal relations? How could one align experimental quantitative approaches with the more idiographic case-based reasoning? Such questions were made more difficult by social and institutional considerations. Liddell frequently complained that disciplinary doctrinal commitments were rigid and difficult to overcome, impervious to the evidence coming from animal experimentalists; one could “publish it, give him a reference, and he still will not believe it” (Liddell in Tanner and Inhelder 1956: 128). Unsurprising, Liddell tended to gravitate to open-minded audiences who shared his multi-disciplinary vision.

Liddell’s relationship with Frank Fremont-Smith, Medical Director of the Josiah Macy Jr. Foundation, was a significant source of support. From the early 1930s, the Macy Foundation generously funded the construction and activities of the Behavior Farm. Recognizing Liddell’s dual expertise in psychology and physiology, Fremont-Smith believed he shared the Foundation’s aspiration to foster cross-disciplinary interactions and understanding. In 1942, he invited Liddell to “a small informal conference on cerebral inhibition” focused on “physiological and psychological mechanisms”.^36^ This meeting served as a platform from which more famous and influential series of conferences followed.^37^ Liddell’s participation in such a series through the 1950s, introduced him to a broad range of work on behavior. Particularly important was the science of the ethology, represented by Konrad Lorenz and Niko Tinbergen. Liddell had been exposed to ethological approaches as early as 1937, Freemont-Smith sending him a newly translated article by Lorenz as “it contained so many interesting observations and thoughts concerning the relationship of instinct and conditioned reflex to behavior”.^38^ When Liddell was joined at Behavior Farm by an ethologist Margaret Altmann, he tasked her with translating papers written by Heini Hediger and Konrad Lorenz.^39^ Subsequently, Liddell worked to build common cause with ethology, inviting both Tinbergen and Lorenz to Cornell on a number of occasions through the 1940s and 1950s.

In 1954, Lorenz visited Cornell to give a series of prestigious lectures on “comparative ethology” (Anon 1954: 3).^40^ Drawing inspiration from comparative anatomy, Lorenz asserted that behavior should be approached by the scientist in the same way—tracing evolutionary lineages through the identification of behavioral structures. These structures were, therefore, homologous. However, within ethology, the promise of behavioral homology still tended to exceed its practical utility. Beyond the classic work of Lorenz on ducks and Niko Tinbergen on herring gulls, there were few rigorous demonstrations. Nevertheless, Lorenz made a persuasive argument for homology as the conceptual underpinning for reasoning from animal to human behavior (cf. Burkhardt 2005: 185). Liddell was taken by the ethological concept of behavioral homology as it suggested neurosis was shared across species through evolutionary lineage and common descent. If animal neurosis was a mere analogy of the human, the relevance of comparative study was diminished. Accordingly, integrating ethology into the work of the Behavior Farm provided a new language and conceptual credibility to their comparative approach. Ethological homology suggested that “experimental neuroses are basically the same for dog, pig, sheep, goat and man”.^41^ Accordingly, Liddell drew on ethology to rebuff criticism:

I do not believe that we have made a liberal use of analogies and thus begged the question of the relationship of experimental neurosis in animals to emotional disorders in man. The emotionally disturbed sheep is not “anxious” and does not suffer from “insomnia” in the clinical sense. It does, however, if one examines its behavior in barn and pasture exhibit the homologues of anxiety and insomnia. The zoological principle of homology need not be limited to structures such as the sheep’s foreleg and the human hand and arm. The ethologists Lorenz and Tinbergen as well as ourselves believe that there are homologous behaviors as well as structures. It is these homologous behaviors in sheep and man that we are seeking to decipher in order to disclose the animal origins of Freudian psychodynamics.^42^

In this way, the ethological language of homology helped Liddell defend the validity of his comparative approach to the study of neurosis.

Equally important, however, were the practical synergies Liddell identified in the shared challenges of the epistemology of observation. Liddell saw ethology as an emergent field struggling with same methodological problem that perplexed him in his own work: how to include intuitive and intimate knowledge of the animal within a scientific study of behavior. An extensive discussion of this problem occurred at the Macy sponsored First Conference on Group Processes in 1955 where both Lorenz and Tinbergen attested to the importance of an intuitive understanding of animal behavior. Responding to a presentation by the American comparative psychologist Daniel S. Lehrman (1955), Tinbergen explained:

We just cannot avoid [intuition] in our study of animals. I might even say that we often begin by looking at a fish as if it were a man. When I see my sticklebacks attack one another, I attack with them. In the beginning I tried to get rid of this kind of intuition entirely. I thought it was unscientific. I now begin to feel that it is so only in a certain sense. But it is indispensable, really, and one must use it, however, only while being completely aware of what one is doing. It gives one ideas that leads to hypotheses. Then comes the next step; that is the checking of ideas. This is the really scientific part (Schaffner 1955: 263).

Intuition in this sense was the scientist’s feeling for the “gestalt”, as Lorenz explained: the scientist knows “there is some sort of regularity in the phenomena observed. He knows there *is* a regularity, long before he knows *what* it is” (emphasis original Schaffner 1955: 262). Moreover, the ability to correctly reason from such intuition increased in line with the ability of observer and observed to communicate. Margaret Mead made this point in contrasting two experiences of studying the same “primitive peoples” set 25 years apart. On her first visit her subjects could not understand her, but on the second, they had “entered the modern world” and fully comprehended her intent. Mead reported getting “a totally different level of observation … comparable to the difference between observing babies and observing children old enough to talk about things, and especially to understand what you are doing” (Schaffner 1955: 265). Lorenz readily agreed explaining how he made “better progress with the analysis of those animals to whom I can ‘say something’ and make myself understood”, which he famously achieved by imitating the behavior of the animals he studied (ibid.). Such discussions played an important role in helping Liddell overcome his “squeamishness about anthropomorphizing”.^43^ Participation in the Macy conferences and his engagement with ethology strengthened Liddell’s resolve to pursue a comparative program of research on animal neurosis.

Accordingly, the 1950s saw Liddell adopt a robust confidence where he sought to directly link his work to psychiatry, rather than expecting others to draw on his research and apply it to man. In 1952, he served as a member of the US Army research project on combat stress in Korea and found similarities between the experimental neurosis in the animal and combat stress in soldiers.^44^ However, it was in the maternal relationship among sheep and goats that Liddell saw his best opportunity to influence psychiatry.^45^ Consistent with his interest in bringing elements of the farm into the laboratory, in the 1940s, Liddell had begun to explore how the presence of another animal influenced the experimental animal’s emotional responses to conditioning. Using the opportunity to promote the “psychobiological survey” of the animal’s entire life situation, Liddell described how his understanding of the mother’s behavior towards their young and the potentially catastrophic consequences of its disruption through birthing complications, turned his attention to the responses of the young and their mothers.^46^ With the aid of Helen Blauvelt, a field naturalist, Ulric Moore, his long-standing research assistant and manager of Behavior Farm, and Moore’s wife Frances, they began an extensive series of experiments with lambs and kids subjected to stress under a variety of conditions—free or restricted activity, with or without mother, varying times after birth, after adoption by new mother—all seeking to understand the mother’s role in protecting her young from the effects of psychic stress. In one form of the experiment, they would remove one twin lamb or kid immediately after birth, whilst the other was allowed to remain with its mother. Aged between 4 h to 2 weeks, both were exposed to a mild electric shock; one alone and one with the mother. Liddell found the presence of the mother protected the lamb or kid from any emotional trauma, allowing the animal to move freely and confidently around the pen. The orphaned animal, in contrast, became “psychologically frozen”^47^: “Here the animal, the little kid, forms a Pavlov frame for himself. He gives up the free exploration of the room… He has made his own harness through his self restraint”.^48^ Having developed chronic neurosis, the young would often die within weeks of birth.

Liddell described his work on mother-young relations as marking a “new phase” in the work of the Behavior Farm, and a critical advance in the development of a comparative psychobiology (Block 1963: 2). It was to Liddell’s work that John Bowlby (1951) first turned when seeking experimental evidence for his theories regarding the significance of maternal deprivation. Liddell, in turn, drew upon René Spitz’s studies of “hospitalism” on child development.^49^ He also established a productive working relationship exploring the parallels between mother-young interactions in humans and goats with the pediatrician Julius Richmond, of the New York State University College of Medicine at Syracuse, who went on to administrate the Head Start educational program, designed to overcome the effects of deprivation (Hersher et al. 1958). Liddell promoted sheep and goats as ideal experimental animals for understanding these processes. They shared the human mother’s unidirectional and individualized affection towards a specific child: “Not all mammals are built with this singlemindedness of purpose”.^50^ In the sheep and goat, vital, synchronous and predictable interactions between mother and offspring involving touch, smell, and feeding, which they characterized as “mutual conditioning”, were concentrated in a single hour immediately following birth.^51^ The characteristics of these interactions would then determine the qualities of later individualized caring. This allowed researchers to observe, compare and manipulate various aspects of mothering behavior and maternal care in a focused and controlled manner, offering lessons “on how to raise human beings”.^52^

In Liddell’s study of maternal care ethology assumed a new prominence operating to integrate activities to form a coherent whole. The conditional reflex remained critical but was no longer the straightforward experimental tool that he had once assumed it to be. Its results were now recognized to be shaped by “facts which lay outside the confines of the laboratory conditioning procedure” (Liddell 1942: 395). The conditional reflex had been redefined as a stress agent, a precise means of testing an animal’s susceptibility and resilience in relation to its developmental history and, thus, of understanding the significance of more fundamental biological and social relationships and the consequences of their disruption. Ethology now contributed a method, and one that encouraged communication with pediatricians through research site visits, clinical demonstrations, and films as they carried out reciprocal observational studies of mothers with human infants, lambs and kids, as they “move move together, easily or with difficulty, in sequences of biological significance”.^53^ It provided theoretical and conceptual strength, allowing Liddell to counter criticisms of analogical reasoning from animal to human by means of a behavioral homology: the shared evolutionary function of mother-neonate *physical* and *emotional* interaction. Finally, it provided the Behavior Farm with an enhanced practical purpose: maternal attachment was a critical biological relationship, of great import to psychiatry and developmental psychology and physiology, and could only be fully understood experimentally through an ethologically-oriented and comparative psychobiology that encompassed both laboratory and field.

## Conclusion: a farm of many fields

In *Emotional Hazards in Animals and Man*, Liddell summarized the thrust of his lifelong research in a breezily written monograph aimed at a broad audience (Liddell 1956).^54^ Opening with “a clinical demonstration”, Liddell invited the reader to “pretend we have assembled for a medical staff meeting” in which he described the responses of a “patient” (a ram named Robert) to a series of experiences with the conditioned reflex (Liddell 1956: 3).^55^ This was a lightly fictionalized but heavily consolidated account of numerous such demonstrations which Liddell (and the ram, Robert, one of his favored demonstration animals) had performed over the years to a diverse audience of interested physiologists, clinical psychiatrists, physicians, behavioral scientists, sociologists, anthropologists and others.^56^ Here, Liddell narrated his everyday working practices, presenting behaviors and responses in the animal “patient” that were readily recognized by anyone familiar with behavioral symptoms of human mental illness. *Emotional Hazards in Animals and Man* proceeded to make a strong case for the mutual benefits of collaborating across disciplines, of forging a comparative psychiatry. In concluding, Liddell wrote:

Our contemporary conformities, with the mechanization of thought and feeling they impose, enhance the baleful operation of the neurotic process in thwarting the strivings of the human spirit. But every individual possesses a secret weapon with which to combat neurosis and gain freedom. That weapon is the creative impulse, which provides vigor and enchantment; buoyancy and elegance; or incisiveness of thought and flexibility of spirit, whichever pair of terms one may choose. Perhaps all should be included. From our point of view, it is this creative impulse which generates zest and insures mental health. (Liddell 1956: 94)

This revealed a symmetry between Liddell’s experimental studies of neurosis and his approach to the intellectual, disciplinary and social arrangement of science. Working across terrains, seeking synergies where others saw irreconcilable difference, reflected Liddell’s refusal to allow restraints such as disciplinary convention obscure creative approaches to potentially shared problems. We can see this in Liddell’s decision to work at the interface of two broad traditions that were commonly pitted against one another. First, Pavlovian conditioning—physiological, experimental and situated in the laboratory. Second, Freudian psychoanalysis—focused on the inner psychic experience of human individuals and situated in the clinic. Rather than conflicting, Liddell saw the two as converging on the same fundamental problem: the functional significance of emotion.

Importantly, Liddell’s eclectic and synthetic approach was anchored in specific problems drawn from and shared by different material and social systems of production. To take one field, medical education, Liddell explained the “student learns to recognize an alarming array of obscure organ dysfunctions only to discover when he becomes a practicing physician that patients come to him in a state of pain and fear[…] An anatomist, plus a physiologist plus a pathologist plus a bacteriologist plus a biochemist does not make a physician”. Regardless of the problem, the physician must first and foremost apply the “sensitivities of naturalist and sociologist in observing the nuances of ordinary human behavior” to act successfully as a “*preventive psychiatrist*” (Liddell 1956: 50). What we see here is how ethology, embodying the sensitivities of naturalistic and sociological observation, provided Liddell with the means to forge a shared way of seeing. With the ethological gaze, Liddell could work comparatively across laboratory and farm. At the same time, ethology ensured that his work could communicate easily with psychiatry and other fields interested in generalized understandings of behavior.

In this sense Liddell’s work contrasts with Kohler’s description of observation and comparison as having become “second-best practices in a landscape dominated by labs. And whole organisms and organisms in situ became less real than their disassembled parts” (Kohler 2002: 3). Comparison was essential to Liddell’s experimental work operating as means and justification for the incorporation of intuitive reasoning into his scientific epistemology. Furthermore, comparison alongside intuitive reasoning allowed the work of the Behavior Farm to study parts but maintain a sense of the whole. In the United States, Pavlov was understood conventionally to have worked to exclude subjective experience from science.^57^ In contrast, as Dr. Thomas M. French observed at the 1937 meeting of the American Psychopathological Association, whilst:

we might all agree with Pavlov’s general feeling that methodologically it had certain advantages to devise a method which made him independent of the subjective impressions … that didn’t necessarily mean he had to entirely isolate himself from the intuition that everyone of us has […] One of the things that most impresses me with Dr. Liddell’s work is the fact that he is correcting this tendency, that is, in his following of Pavlov’s method, he is correcting this tendency to observe only one single, slight response that is characteristic of Pavlov’s reported work and he is observing the behavior of the animal as a whole. The result is that the behavior of the experimental animal again becomes humanly intelligible to us instead of leading us into a realm of reactions that takes careful and detailed analysis to bring back into the relationship with clinical observation (discussion in Liddell 1938: 1042–1043).

For Liddell, intuitive comparative reasoning served a twofold purpose. On the one hand, it was an essential guide allowing him to ‘turn to the end to see how it comes out’ in the manner that a curious reader might flick to the end of a detective novel. On the other, by sustaining a productive role for subjective experience within the laboratory space, Liddell’s intuitive comparative reasoning provided a bridge for communication between experimental and clinical communities. It operated as the medium *and* justification for drawing on his own experience to intuitively interpret and predict the experience of his experimental animals.

Realizing a new psychobiology of emotional disorder in practice was, however, far from straightforward. From his very first experiments in thyroidectomy, Liddell had to continuously pause, reflect, and reinterpret unexpected and unpredictable animal behavior. New technical innovations, most notably the Pavlovian reflex technique, were adopted and adapted to contain the unexpected, rendering it productive. However, the more Liddell refined the conditioned reflex approach, building soundproof cameras, two-way windows, automated recording and stimulating devices and so effectively mechanizing the production of neuroses in his growing flock, the more he found himself grappling with the unpredictable and often intractable behavior of his experimental animals. It was for this reason that legitimizing intuitive reasoning from the observation of individuals became so important to Liddell. Although Pavlovian conditioning and other techniques promised a certain kind of objectivity associated with quantification and experimental control, reconciling this promise with the experience of unexpected behavioral responses relied on knowledge gained through intimate relationships more akin to the case-based knowledge of the clinician. Liddell captured the complex interdependency of experimental measurement and ethological observation, part and whole, in describing the reflex as the *vade mecum* of experimental psychiatry. The literal meaning of *vade mecum*, shorthand for a reference book or useful object regularly carried by a person and used as a guide, is “walk with me”, which evokes the importance of intimate knowledge of the individual in the interpretation of behavior. Such knowledge was gained not merely by studying animals in the laboratory but understanding the individual and their (natural) history in a more generalized sense.

Liddell’s approach to the study of behavior made a virtue of tensions and contradictions whether these were found in the disciplines he worked across, the tools and concepts he sought to apply, or the people and communities he brought together. Ethology encouraged Liddell to extend his comparative gaze to the barnyard and pastures of the farm, comprehending pathological behavior not only in relation to ‘normal’ individuals but also within ‘normal’ environments. Though this extended gaze, animals were observed to carry their responses to the forced drills of the Pavlovian method into the ‘natural’ environment of the barnyard—understood to be their ‘normal’ life world. As such, their experience in the laboratory was shown to have stripped them of their ability to adapt as autonomous, creative and active individuals to new life situations. In this way, the work of the farm was gradually expanded over time. Initially intended as a mechanism to produce and maintain sufficient quantities of animals to meet the experimental needs of the laboratory, the farm evolved to form part of the experimental landscape. Extending the comparative gaze and following animals outside to the farm allowed Liddell to demonstrate that pathologies generated by the conditional reflex were permanent “scars upon the personality or brain”.^58^ It followed that behavioral pathologies were only meaningful when they considered “the animal as an organism that has continuity from birth to death and that has an interrelationship with other members of its animal world that determines its basic functioning as an individual”.^59^ Thus normal and pathological behavior could only be understood in the context of an individual’s continued ability to adapt successfully to their environment.^60^

In emphasizing the observation of the whole organism and the understanding of individual behavior, ethology also provided a shared conceptual and theoretical repertoire. This allowed Liddell’s work to travel to, and be understood within, psychiatry and clinical therapy. Detailed observation of animals throughout their life-spans held the potential for remaking psychiatry into a properly experimental and scientific field in a way that was not possible in the study of human subjects alone. Liddell found the ethological language of ‘homology’ to be particularly flexible and useful in his attempts to extend to the human the processes of emotional breakdown in his sheep and goats. At the same time, engagement with ethology encouraged Liddell to refocus his work around the study of mother-young relations. Moving away from experimental neurosis also helped Liddell circumvent growing criticism of what Karl Lashley described as ‘homologies of animal and human neuroses’, which gained traction in the 1940s.^61^ As such, the conditioned reflex was refashioned as a catastrophic and generalized stressor used to understand the homology of mother-neonate interaction. Accordingly, the complex interactions between social and biological processes at play on the farm were skillfully reworked. Poised at it was on the border between laboratory and field, the Behavior Farm provided a critically important exploratory space for this inter-disciplinary work, and Liddell integrated the study of behavior, physiology and psychiatry into a scientifically valid, flexible and purposeful comparative psychobiology. He created a farm of many fields, a hybrid space that facilitated the movement of ethological ideas and methods into experimental psychopathology.

## Footnotes

1 See Todes (2014), for a detailed analysis of Pavlov’s experiments relating to neurosis.

2 Howard S. Liddell, Evidence of interrelation between doctrines, manuscript, p. 5. Howard S. Liddell Papers,#14-23-1466, Box 3, Folder 9. Division of Rare and Manuscript Collections, Cornell University Library (hereafter HSL).

3 Liddell was not alone in his attempts to do so. Neal E. Miller (1939), for instance, investigated Freudian psychodynamic concepts such as displacement of aggression in rats, while Jules Masserman (1943) sought to provide psychoanalysis with an objective basis through Pavlovian methods applied to the study of cats (cf. Winter 2016).

4 Influential work within ontological hybridity includes Bruno Latour (1993), Sarah Whatmore (2002), and Donna Haraway (2003).

5 In reviewing Kohler, Gregg Mitman (2003) has also questioned Kohler’s focus on the movement of instruments and methods as decidedly one way, from lab to field.

6 For another analysis of the ways in which field influenced the laboratory, see Sharon E. Kingsland (2009).

7 For an overview of Simpson’s work see the memorial edition of *Quarterly Journal of Experimental Physiology*, 17, 1927, which includes within its tributes by Simpson’s students a contribution by Liddell.

8 An additional bonus to these species was that the wool and mutton of the control helped defray the cost of experiment.

9 Apparent as, in later years, he returned to and re-interpreted the (now significant) labyrinth observations through the lens of more developed behavioral theories such as “vigilance”. See Liddell (1954).

10 It is notable that in his original reports of the 1920s, Liddell refrained from using anthropomorphic language such as “ambition” and was relatively restrained in his comparison of human and animal.

11 H. S. Liddell, “Psychobiological analysis of emotional behavior in animals under stress of conditioning from birth to maturity”, WHO Study Group on Psychobiological Development of the Child, 13 November, 1953., HSL, Box 3, Folder 9; H. S. Liddell and Clive M. McCay, Progress Report for Research Grant MH 41/C, July 1, 1952, HSL, Box 6, Folder 21.

12 H. S. Liddell, in conference transcripts, untitled and undated, HSL, Box 5, Folder 36.

13 H. S. Liddell “The biology of individual and group prejudice”, n.d. c. 1950s, HSL, Box 5, Folder 40.

14 H. S. Liddell, “Experimental analysis of stress and individual self-control in animal behavior”, n.d., HSL, Box 5, Folder 51.

15 Letter, Liddell to Edmund Day, President of Cornell University, January 30, 1942, HSL, Box 1, Folder 45.

16 H. S. Liddell to Frank Fremont-Smith, April 7, 1949, HSL, Box 5, Folder 50.

17 H. S. Liddell, in conference transcripts, op. cit.; Liddell, A Biological basis for psychopathology, Presidential Address, American Psychopathological Association, 1957, p. 3, HSL, Box 5, Folder 39.

18 H. S. Liddell and C. M. McCay Progress report, 1952, op. cit., p. 11.

19 H. S. Liddell, A new manifestation of experimental neurosis in sheep and goat, June 1944, HSL, Box 1, Folder 28.

20 Liddell, “A Biological basis”, op. cit., p. 7.

21 Ibid., p. 8.

22 Ibid.

23 H. S. Liddell and C. M. McCay Progress report, 1952, op. cit., p. 6.

24 H. S. Liddell to Henry Murray, Department of Social Relations, Harvard, May 23, 1957, HSL, Box 2, Folder 20.

25 Notes taken from October 2 to November 1, 1958, Behavior Farm Lab, Jalal Besharat, HSL, Box 5, Folder 19.

26 H. S. Liddell to Karl Lashley, November 7, 1945, HSL, Box 2, Folder 17.

27 Ibid.

28 Study Group on Psychobiological Development of the Child Proceedings of the Second Meeting, London, 1954, conference transcript of Liddell comments, HSL, Box 1, Folder 13, p. 3.

29 H. S. Liddell, Stress, emotion, and mental health, Annual Lecture, 1954, National Institutes of Health, HSL, Box 5, Folder 39, p. 13.

30 See Dror (1999) for an analysis of the various ways physiologists sought to contain and control emotion among their animals in their laboratory practices.

31 This tension is evident in an early overview of work at the Behavior Farm; see Anderson and Parmenter (1941).

32 Emphasis original. Liddell “The biology of individual”, p. 15.

33 For instance Cobb was an invited guest to the Macy sponsored First Conference on Group Processes which Liddell organized at Cornell in September 1954. See HSL, Box 3, Folder 2. Cobb was also a keen amateur naturalist with a particular interest in bird behavior. See White et al. (1988).

34 Howard S. Liddell “The Therapeutic Challenge of Pavlovian Conditioning and Experimental Neurosis in Animals”, n.d. c. 1950s, p. 1, HSL, Box 5, Folder 41.

35 Ibid. pp. 7–8, emphasis in original. Liddell went so far as to suggest that if Freud had visited the farm to acquaint himself with “the facts” of comparative psychopathology, he would surely have controverted his own claim that neurosis was an exclusively human condition.

36 Letter, Frank Fremont-Smith to Howard S. Liddell, April 8, 1942, HSL, Box 1, Folder 54. Attendees to the 1942 meeting included Gregory Bateson, Frank Beach, Arturo Rosenblueth, Milton H. Erikson, Lawrence Kubie, Jules H. Massermann, Margaret Mead, Warren S McCulloch, John Whitehorn and Harold G. Wolff—leaders in their respective fields.

37 Liddell’s role in the 1942 Macy sponsored meeting on cerebral inhibition is rarely noted but see Steve Joshua Heims (1993: 14–18). For the history of Macy conferences in relation to cybernetics see Ronald R. Kline (2015).

38 The manuscript was titled “The companion in the environment of the bird”. See Letter, Frank Fremont-Smith to Howard S. Liddell, April 28, 1937, HSL, Box 1, Folder 54. The archives show a gradual intensification of his interest in Lorenz’s work through the 1940s to a high point in the 1950s.

39 Letter, H. S. Liddell to Robert Lambert of the Rockefeller Foundation, January 17, 1941, HSL, Box 2, Folder 17; Ulric Moore to Dr F. Brunner, March 16, 1960, HSL, Box 5, Folder 23. See also, Box 5, Folders 16 and 17.

40 That Lorenz had also studied animals under semi-captive conditions at his home, constantly observing their behavior, undoubtedly increased the relevance of his ideas and methods to Liddell’s farm. On this and the relation of place to the field of ethology more generally, see Richard Burkhardt (1999: 500) and see also Burkhardt (2005).

41 H. S. Liddell “The Therapeutic Challenge of Pavlovian Conditioning and Experimental Neuroses in Animals", HSL, Box 5, Folder 41, p. 7.

42 Liddell, manuscript, untitled response to criticism from Dr. Bloomberg and Dr. Foley, p. 1, n.d. c.1950s, emphasis in original, HSL, Box 5, Folder 43.

43 Liddell “Study Group”, op. cit., p. 3.

44 Howard Scott Liddell, Memorial Address, October 31, 1962, Anabel Taylor Chapel, by Frank S. Freeman, HSL, Box 1, Folder 14. See Long (2014).

45 Liddell “The biology of individual”, op. cit.

46 Liddell, “Stress, emotion, and mental health”, op. cit.

47 Liddell, quoted in Bowlby (1951, p. 21).

48 Liddell in conference transcripts, op. cit.

49 Liddell to Spitz, November 27, 1946, HSL, Box 2, Folder 46. See Spitz (1945).

50 L. Hersher, J. B. Richmond, A. U. Moore, Some Factors Affecting Maternal Care in Sheep and Goats, read at Orthopsychiatry, 1960. HSL, Box 5, Folder 30.

51 H. S. Liddell, in conference transcripts, op. cit.

52 A. U. Moore to Mrs A. A. Lund, December 16, 1962, HSL, Box 2, Folder 17.

53 Helen Blauvelt, untitled paper, May 9, 1956, HSL, Box 5, Folder 20.

54 In recognition of the Macy Foundation’s role in making possible the unbroken continuity of his research Liddell framed this work as a “testimonial to the inspiration of an eminent catalyst of interdisciplinary research in the medical sciences, Dr. Frank Fremont-Smith” (Liddell 1956: x).

55 In her study of attachment theory, Marga Vicedo writes: “Contrary to Bowlby’s own assertions that attachment theory was based on the convergence of results from investigations about infants, birds, and monkeys, I argue that the theory’s success was due to the cross-disciplinary alliance between Bowlby and Lorenz” (2013: 4). In Liddell’s demonstrations, a convergence of results across species was apparent and could, within the constraints of the time, be witnessed. One prominent witness was Bowlby, who visited the Behaviour Farm after the publication of *Maternal Care and Mental Health* (see letter, Bowlby to Liddell, May 5, 1953, HSL, Box 1, Folder 33). Whilst cross disciplinary alliances were no doubt as useful to Bowlby as they were to Liddell, it is nonetheless important to recognise that such alliances were grounded in experimental work—not least Liddell’s, who is absent from Vicedo’s analysis.

56 Though demonstrations more usually occurred on the Behavior Farm Liddell occasionally travelled to other medical schools. In November 1954, for instance, Liddell travelled with Robert the ram and a goat named ‘Brown Billy” to conduct demonstrations at the University of Pittsburgh School of Medicine. This was on the invitation of Professor Henry W. Brosin who had been impressed on visiting the Behavior Farm with Liddell’s substitution of goats for patients. Such trips demonstrated a further advantage of Liddell’s choice in species, as sheep and goats all travelled well and made little noise and few demands. See correspondence, HSL, Box 3, Folder 8.

57 A far more nuanced account of Pavlov’s changing engagement with subjective experience is provided by Daniel Todes (2014).

58 Liddell, “Stress, emotion”, op. cit.

59 Draft of a grant application, untitled, 1955, HSL, Box 7, Folder 13.

60 There is a striking similarity between Liddell’s approach to the relation between the normal and pathological and Georges Canguilhem’s mediations—the individual being neither diseased nor healthy in an absolute sense, but in a relational sense, as an autonomous being adapting and maintaining “its own vital norms”, in a given environment (Canguilhem 2008: 113). See also Canguilhem (1991).

61 Karl Lashley to Liddell, October 26, 1945, HSL, Box 2, Folder 17.
